# Liver Inclusion Appears to Be Protective Against Graft Loss-Due-to Chronic But Not Acute Rejection Following Intestinal Transplantation

**DOI:** 10.3389/ti.2023.11568

**Published:** 2023-09-14

**Authors:** Rodrigo Vianna, Jeffrey J. Gaynor, Gennaro Selvaggi, Ahmed Farag, Jennifer Garcia, Akin Tekin, Marina M. Tabbara, Gaetano Ciancio

**Affiliations:** ^1^ Department of Surgery, Miami Transplant Institute, Miller School of Medicine, University of Miami, Miami, FL, United States; ^2^ Department of Surgery, Zagazig University School of Medicine, Zagazig, Egypt; ^3^ Department of Pediatrics, Miami Transplant Institute, Miller School of Medicine, University of Miami, Miami, FL, United States

**Keywords:** intestinal transplantation, graft loss-due-to acute rejection, graft loss-due-to chronic rejection, prognostic factors, long-term results CHR, chronic rejection

## Abstract

In intestinal transplantation, while other centers have shown that liver-including allografts have significantly more favorable graft survival and graft loss-due-to chronic rejection (CHR) rates, our center has consistently shown that modified multivisceral (MMV) and full multivisceral (MV) allografts have significantly more favorable acute cellular rejection (ACR) and severe ACR rates compared with isolated intestine (I) and liver-intestine (LI) allografts. In the attempt to resolve this apparent discrepancy, we performed stepwise Cox multivariable analyses of the hazard rates of developing graft loss-due-to acute rejection (AR) vs. CHR among 350 consecutive intestinal transplants at our center with long-term follow-up (median: 13.5 years post-transplant). Observed percentages developing graft loss-due-to AR and CHR were 14.3% (50/350) and 6.6% (23/350), respectively. Only one baseline variable was selected into the Cox model indicating a significantly lower hazard rate of developing graft loss-due-to AR: Transplant Type MMV or MV (*p* < 0.000001). Conversely, two baseline variables were selected into the Cox model indicating a significantly lower hazard rate of developing graft loss-due-to CHR: Received Donor Liver (LI or MV) (*p* = 0.002) and Received Induction (*p* = 0.007). In summary, while MMV/MV transplants (who receive extensive native lymphoid tissue removal) offered protection against graft loss-due-to AR, liver-containing grafts appeared to offer protection against graft loss-due-to CHR, supporting the results of other studies.

## Graphical Abstract

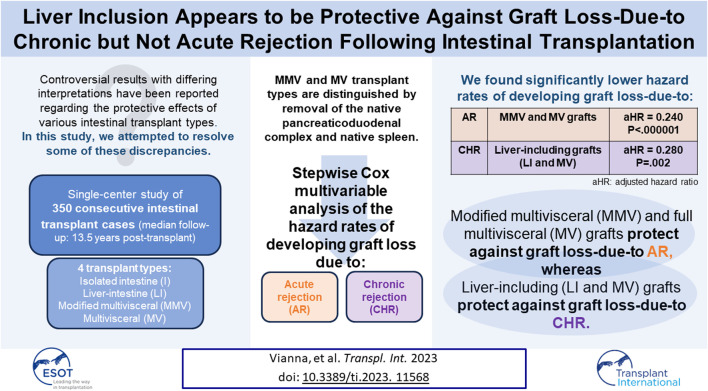

## Introduction

In intestinal transplantation controversial results with differing interpretations on the protective effects of various transplant types have been reported, with liver-including grafts being shown in some studies to have significantly more favorable graft survival [1–4] and lower graft loss-due-to rejection [5–7] rates. However, other studies, have shown that modified multivisceral (MMV) and full multivisceral (MV) transplant recipients have significantly more favorable freedom from acute cellular rejection (ACR) [8], freedom from severe ACR [8–12], and lower graft loss-due-to rejection [8, 12–14] rates in comparison with isolated intestine (I) and liver-intestine (LI) transplant recipients. The latter results suggest that there is a protective effect of MMV and MV which is likely explained by more extensive native lymphoid tissue removal.

In our recent report of 445 consecutive intestinal transplant cases [8], 76.8% (53/69) of the observed graft losses-due-to rejection (during the first 60 months post-transplant) were due to acute rejection (AR), with 23.2% (16/69) being due to chronic rejection (CHR). In contrast, among the 101 observed graft losses-due-to rejection (out of a total of 500 intestinal transplant cases) reported by the University of Pittsburgh [6], only 25.7% (26/101) were due to AR, whereas 74.3% (75/101) were due to CHR. Reported follow-up was much longer in the latter study. In addition, it was clearly reported in Abu-Elmagd et al [6] as well as in an earlier University of Pittsburgh report [15] that the hazard rate of graft loss-due-to CHR was highly significantly lower among recipients of liver-containing (LI and MV) grafts in comparison with liver-free (I and MMV) grafts.

In a separate Abu-Elmagd et al study [16], the hazard rate of developing chronic (but not acute cellular) rejection was significantly higher among patients 1) with preformed donor specific antibodies (DSAs) that persisted over time post-transplant or 2) who developed *de novo* DSAs post-transplant. Patients with liver-containing grafts were significantly less likely to develop either persistent or *de novo* DSAs in that study [16]. In addition, Wu et al [17] showed that the presence of DSAs was associated with a significantly higher risk of the patient developing acute antibody mediated rejection (AMR), and liver-containing allografts offered significant protection against the development of acute AMR.

We recently reported the results of a rather comprehensive multivariable analysis of prognostic factors for the hazard rates of developing a 1st ACR, a severe ACR, and graft loss-due-to rejection (AR or CHR) during the first 60 months post-transplant (among 445 consecutive intestinal transplant cases at our center between 1994–2017); however, separate analyses of predictors of the hazard rates of graft loss-due-to AR vs. CHR had not been performed [8]. In the attempt to resolve some of the previously reported discrepant results between our center and those of other centers, we wanted to analyze multivariable predictors of the hazard rates of graft loss-due-to AR vs. CHR in our cohort with follow-up longer than 60 months post-transplant. We therefore analyzed all consecutive intestinal transplants performed at our institution between 1994 and 2012 (350 cases), with a date of last follow-up of 15 March 2019 (thus, a planned minimum follow-up of over 6 years post-transplant). Results of this observational study are presented here.

## Materials and Methods

### Patients and Immunosuppression

Our historical cohort of 350 consecutive intestinal transplant cases (308 primary recipients and 42 retransplants) at the Miami Transplant Institute during 1994–2012 were followed prospectively through 15 March 2019—the same last follow-up date as in our recent reports [8, 18]. In order to allow for a sufficiently long minimum follow-up of all patients, our more recent group who were transplanted at our center since 2013 were excluded here. Over the years the center institutional review board approved each immunosuppression protocol used for these patients; all patients gave written informed consent before enrollment. In addition, all clinical and research activities adhered to the ethical principles (as revised in 2013) of the Helsinki Declaration.

As in our previous reports [8–12, 18, 19], recipients were divided into four transplant types: isolated intestine (I), liver-intestine (LI), modified multivisceral (MMV), and multivisceral (MV). While the donor pancreas was sometimes transplanted into I and LI recipients, the native pancreaticoduodenal complex was always left intact along with the native spleen (in the great majority of cases). Conversely, MMV and MV transplants were defined by removal of the native pancreaticoduodenal complex and native stomach, along with performing a native splenectomy (in the great majority of cases). In addition, the intent with MMV and MV transplants was to orthotopically transplant *en bloc* the donor stomach, donor pancreaticoduodenal complex, and donor intestine into the recipient [8–12, 18, 19]. Since a near-total removal of the gastrointestinal tract (except for a segment of large intestine), including native splenectomy, is performed in MMV and MV recipients, a much more complete lymphadenectomy is achieved compared with I and LI grafts, where splenic, celiac, and gastric lymph nodes are left *in situ* [8–12, 18, 19].

Recipients were divided into four induction groups [8]. Group 1 (1994–1997) comprised 44 recipients who received no/old induction therapy (high-dose corticosteroids only in 34, OKT3 in 7, and cyclophosphamide in 3). Among primary cases, OKT3 was used first (8/94–1/95), followed by cyclophosphamide (4/95–6/95). Once their use was abandoned, high-dose corticosteroids only were used (7/95–12/97). Group 2 (1998–2011) comprised 159 recipients who received an anti-CD25 monoclonal antibody (daclizumab in 156, and basilixmab in 3). Daclizumab (2 mg/kg) was given on postoperative days 0, 7, and 14, and then every 2 weeks during the first 3 months post-transplant; thereafter, daclizumab dose was reduced to 1 mg/kg every 2 weeks for the following 3 months and then stopped. Basiliximab (10 mg) was given on postoperative days 0 and 4, as the three recipients were small children (<35 kg). Group 3 (2001–2011) comprised 113 recipients who received alemtuzumab, with two different schedules being used: 0.3 mg/kg ×4 (pre-operatively, immediately post-transplant, and on postoperative days 3 and 7); and 30 mg ×2 (on postoperative days 1 and 4). Group 4 (2006–2012) comprised 34 recipients who were scheduled to receive 3 rATG doses (total planned rATG dose: 5 mg/kg, with 2.0 mg/kg being given on postoperative day 0, and 1.5 mg/kg being given on postoperative days 2 and 4). However, the actual number of rATG doses that these patients received was uneven: 12/34 received only the first dose, 3/34 patients received only two doses, and 19/34 patients received all 3 doses.

Of note, daclizumab was the only induction agent that was used during the 3 year period from 1998 to 2000. Thus, prior to 2001, the various induction approaches were tried sequentially. In 2001, alemtuzumab was introduced as a tolerance induction protocol; however, due to its initially poor results in young children, starting in August 2002, its use was limited to patients 4 years of age or older at the time of transplant [11]. Since August, 2002, most of the patients who received daclizumab induction (Group 2) were children, whereas most of the patients who received alemtuzumab induction (Group 3) were adults. In total, the percentage of adults in Groups 2 and 3 was 15.1% (24/159) vs. 74.3% (84/113), respectively. In addition, only 3/159 of Group 2 patients were transplanted since 2009 (3 young children who received basiliximab); thus, most of the children transplanted during 2009–2011 belonged to Group 4.

Maintenance immunosuppression consisted of TAC and corticosteroids (tapered off by 6–9 months post-transplant) except in patients who received alemtuzumab induction (Group 3), where TAC alone was planned to be used. Target TAC trough levels during the first 3 months and beyond 3 months post-transplant were 15–20 ng/mL and 10–15 ng/mL for patients transplanted during 1994–1997, and 12–16 ng/mL and 8–12 ng/mL for patients transplanted during 1998–2012.

### Clinical Outcomes

Schedules for monitoring, diagnosis, and treatment of ACR episodes and non-immunosuppressive prophylactic therapy have been described elsewhere [8]. Of note, once an ACR was clinically suspected, an immediate endoscopy and biopsy were performed. All ACR episodes were clinically suspected, pathologically diagnosed [20, 21], and treated; ACR grade (mild, moderate, or severe) was determined as the maximum pathologic grade observed during that episode [8, 12]. High-dose corticosteroids (via intravenous bolus injections) were used to treat mild ACR episodes. Antilymphocyte therapy was used in treating steroid-resistant and moderate-to-severe ACR episodes. Graft dysfunction due to resistant rejection was treated with graft removal and listing for re-transplantation.

Graft loss was defined as the date of intestinal graft failure (graft removal) or death, whichever occurred first, with the underlying cause of (triggering event leading to) graft loss being determined in each case [8, 9, 13]. CHR was determined at the time of graft explant based upon conventional pathological criteria [20, 22]. Thus, in contrast to the determination of ACR episodes (as described above), CHR was only determined at the time of graft explant.

### Statistics

Frequency distributions were determined for baseline categorical variables; the mean along with standard error (SE) (as well as the median and interquartile range) were calculated for baseline continuous variables. Tests of association among baseline variables were performed using Pearson (uncorrected) chi-squared tests and ordinary (two sided) t-tests.

Two distinct clinical outcomes were analyzed in this study: graft loss-due-to AR and graft loss-due-to CHR. Differences in freedom from occurrence of each clinical outcome were compared by the log-rank test, with actuarial estimates and time-to-cause-specific failure curves generated using the Kaplan-Meier method. Patients were censored at the time of graft loss from other causes (or at the time of being lost to follow-up, if it occurred). *p*-values ≤ 0.05 were considered to be statistically significant.

Stepwise Cox regression was utilized to identify significant multivariable predictors for each of the two primary outcomes: the hazard rate of developing graft loss-due-to AR, and the hazard rate of developing graft loss-due-to CHR. Again, in performing each analysis, any competing events (i.e., graft losses) occurring other than the cause of interest were treated as censored observations. Baseline variables that were considered for their prognostic value included demographics, transplant-related information, and type of induction received (see Table 1). For two baseline variables in which a small subset of patients had a missing value, the observed mean was imputed for missing values in the multivariable analyses [23]. Testing the validity of the Cox model proportional hazards assumption was performed by considering the inclusion of time by covariate interaction effects.

**TABLE 1:** Distributions of selected baseline variables (*N* = 350).

Baseline variable	Mean ± SE if continuous; percentage with characteristic if categorical
Date of Transplant	Median = 4/1/03; Interquartile Range: 8/1/00–12/15/06
Recipient Age (years)	16.4 ± 1.0 (*N* = 350) Median = 6.9; Interquartile Range: 0.3–65.6
Recipient Age (years):	
<5	46.9% (164/350)
5–17	13.7% (48/350)
≥18	39.4% (138/350)
Recipient Gender:	
Female	49.7% (174/350)
Male	50.3% (176/350)
Recipient Race/Ethnicity	
White (non-Hispanic)	68.0% (238/350)
Black (non-Hispanic)	16.9% (59/350)
Hispanic	13.4% (47/350)
Asian	1.7% (6/350)
CMV Status	
D-/R-	28.0% (98/350)
D-/R+	19.4% (68/350)
D+/R-	24.9% (87/350)
D+/R+	27.7% (97/350)
Donor Age (yr)	10.2 ± 0.7 (*N* = 329) Median: 5.0; Interquartile Range: 0.8–17.0
Intestinal Transplant Status	
Primary	88.0% (308/350)
Retransplant	12.0% (42/350)
Transplant Type:	
Isolated Intestine (I)	27.4% (96/350)
Liver-Intestine (LI)	10.9% (38/350)
Modified Multivisceral (MMV)	9.7% (34/350)
Multivisceral (MV)	52.0% (182/350)
Underwent Native Splenectomy	
No	38.0% (133/350)
Yes	62.0% (217/350)
Native Pancreaticoduodenal Complex Removed	
No	38.3% (134/350)
Yes	61.7% (216/350)
Received a Kidney:	
No	90.9% (318/350)
Yes	9.1% (32/350)
Received a Large Bowel:	
No	53.1% (186/350)
Yes	46.9% (164/350)
Received a Liver:	
No	37.1% (130/350)
Yes	62.9% (220/350)
Received a Pancreas:	
No	32.3% (113/350)
Yes	67.7% (237/350)
Received a Spleen:	
No	74.9% (262/350)
Yes	25.1% (88/350)
Received a Stomach:	
No	39.1% (137/350)
Yes	60.9% (213/350)
In Hospital (vs. at Home) Prior to Transplant	
No	54.9% (180/328)
Yes	45.1% (148/328)
Induction Type:	
Received No/Old Induction^a^	12.6% (44/350)
Received Anti-CD25	45.4% (159/350)
Received Alemtuzumab	32.3% (113/350)
Received rATG (pre-2013)	9.7% (34/350)

Abbreviations: anti-CD25, anti-Interleukin-2 receptor alpha chain (Daclizumab or Basiliximab); rATG, rabbit anti-thymocyte globulin (Thymoglobulin).

a In this subgroup of 44 recipients, 7/44 recieved induction with OKT3, 3/44 received induction with cyclophosphamide, and 34/44 received only high-dose corticosteroids.

## Results

### Baseline Characteristics

Baseline characteristics are shown in Table 1. Median date of transplant was 1 April 2003 (interquartile range: 1 August 2000–15 December 2006). Mean age at transplant was 16.4 years (median age: 6.9 years), with African-Americans and Hispanics comprising 16.9% (59/350) and 13.4% (47/350), respectively; retransplant cases comprised 12.0% (42/350). The percentage of recipients who received isolated intestine (I), liver-intestine (LI), modified multivisceral (MMV), and full multivisceral (MV) allografts was 27.4% (96/350), 10.9% (38/350), 9.7% (34/350), and 52.0% (182/350), respectively. Thus, only 28.4% (38/134) of I/LI grafts vs. 84.3% (182/216) of MMV/MV grafts were liver-containing (*p* < 0.000001).

Crosstabulations of transplant type with the removal of native organs/receiving donor organs are shown in Table 2. The native pancreaticoduodenal complex was removed in 0.0% (0/134) of I/LI vs. 100% (216/216) of MMV/MV cases (*p* < 0.000001). Similarly, native splenectomy was performed in only 3.0% (4/134) of I/LI recipients vs. in 98.6% (213/216) of MMV/MV recipients (*p* < 0.000001). Of note, in 2 I cases with a native splenectomy, these two cases were retransplants of previously failed MV grafts (i.e., native splenectomy was performed during the primary MV transplant). Thus, removal of the native pancreaticoduodenal complex and native splenectomy were jointly performed in only 3.0% (4/134) of the I/LI cases vs. 98.6% (213/216) of the MMV/MV cases (nearly a complete one-to-one relationship). Lastly, the donor spleen was transplanted in no I/LI cases vs. 40.7% (88/216) of MMV/MV cases (*p* < 0.000001). Of note, while extremely rare, 1.6% (3/182) of the MV cases did not receive a donor stomach (documented poor quality in one case).

**TABLE 2:** Cross-tabulations of transplant type with removal of native organs (No/Yes) and receiving donor organs (No/Yes).

Organ-specific surgery	Transplant type			
	I	LI	MMV	MV
Native PC Removed	0.0% (0/96)	0.0% (0/38)	100.0% (34/34)	100.0% (182/182)
Native Splenectomy	2.1% (2/96)	5.3% (2/38)	94.1% (32/34)	99.5% (181/182)
Received a Kidney	3.1% (3/96)	2.6% (1/38)	11.8% (4/34)	13.2% (24/182)
Received a Large Bowel	35.4% (34/96)	21.1% (8/38)	52.9% (18/34)	57.1% (104/182)
Received a Liver	0.0% (0/96)	100.0% (38/38)	0.0% (0/34)	100.0% (182/182)
Received a Pancreas	1.0% (1/96)	52.6% (20/38)	100.0% (34/34)	100.0% (182/182)
Received a Spleen	0.0% (0/96)	0.0% (0/38)	41.2% (14/34)	40.7% (74/182)
Received a Stomach	0.0% (0/96)	0.0% (0/38)	100.0% (34/34)	98.4% (179/182)

Abbreviations: I, isolated intestine; LI, liver-intestine; MMV, modified multivisceral; MV, multivisceral; PC, pancreaticoduodenal complex.

Selected associations among the major baseline characteristics are presented in Table 3. The distribution of transplant type by induction type and by transplant date (before vs. after 1/1/01) shows that LI was much more commonly performed prior to 1 January 2001, whereas MV transplants were more commonly performed since that time (*p* < 0.000001). However, the percentage of patients having liver inclusion (LI or MV) has not changed over time (*p* = 0.32), nor has the percentage of transplanted adults changed over time (*p* = 0.50). Lastly, the distribution of recipient age by induction type shows that anti-CD25 and rATG induction were used mostly in children, whereas alemtuzumab was used mostly in adults (*p* < 0.000001).

**TABLE 3:** Selected associations among the major baseline characteristics.

A) Cross-tabulation of transplant type by induction type					
Transplant Type	Received type No/Old induction	Received Anti-CD25	Received Alemtuzumab	Received rATG (pre-2013)	*p*-value
I	22.7% (10/44)	20.8% (33/159)	40.7% (46/113)	20.6% (7/34)	
LI	34.1% (15/44)	12.6% (20/159)	2.7% (3/113)	0.0% (0/34)	
MMV	0.0% (0/44)	3.8% (6/159)	22.1% (25/113)	8.8% (3/34)	
MV	43.2% (19/44)	62.9% (100/159)	34.5% (39/113)	70.6% (24/34)	
Total	44	159	113	34	< 0.000001

B) Cross-tabulation of transplant type by date of transplant					
Transplant Type	DOT < 1/1/01	DOT ≥ 1/1/01	*p*-value		
I	28.9% (28/97)	26.9% (68/253)			
LI	29.9% (29/97)	3.7% (9/253)			
MMV	4.1% (4/97)	11.9% (30/253)			
MV	37.1% (36/97)	57.7% (146/253)			
Total	97	253	<0.000001		

C) Cross-tabulation of liver inclusion by date of transplant					
Liver Inclusion	DOT <1/1/01	DOT ≥ 1/1/01	*p*-value		
No	33.0% (32/97)	38.7% (98/253)			
Yes	67.0% (65/97)	61.3% (155/253)			
Total	97	253	0.32		

D) Cross-tabulation of recipient age by date of transplant					
Recipient Age (yr)	DOT < 1/1/01	DOT ≥ 1/1/01	*p*-value		
<18	57.7% (56/97)	61.7% (156/253)			
≥18	42.3% (41/97)	38.3% (97/253)			
Total	97	253	0.50		

E) Cross-tabulation of Recipient Age by Induction Type					
Recipient Age (year)	Received No/Old Induction	Received Anti-CD25	Received Alemtuzumab	Received rATG (pre-2013)	*p*-value
<18	59.1% (26/44)	84.9% (135/159)	25.7% (29/113)	64.7% (22/34)	
≥18	40.9% (18/44)	15.1% (24/159)	74.3% (84/113)	35.3% (12/34)	
Total	44	159	113	34	<0.000001

### Graft Loss-Due-to AR vs. CHR

As of the last follow-up date (15 March 2019), the observed incidence of graft loss-due-to any cause was 77.4% (271/350), with the underlying cause of graft loss being due to AR, CHR, infection, and other causes in 14.3% (50/350), 6.6% (23/350), 23.1% (81/350), and 33.4% (117/350) of cases, respectively. Thus, among the transplanted cases who experienced graft loss-due-to rejection, 68.5% (50/73) vs. 31.5% (23/73) were due to AR vs. CHR. The observed percentages of graft loss-due-to AR and graft loss-due-to CHR cases who previously experienced a severe ACR episode were 94.0% (47/50) and 56.5% (13/23), respectively. Median time to graft loss-due-to AR and median time to graft loss-due-to CHR (among the 50 and 23 patients who experienced those events) were 2.3 (range: 0.3–97.8) and 52.9 (range: 3.1–188.3) months post-transplant, respectively. Median follow-up among 79 transplant cases who were still alive with a functioning graft as of last follow-up was 161.1 (range: 79.7–286.5) months post-transplant. Lastly, the total risk set of this 350-patient cohort who were being followed beyond 1, 3, 6, 9, 12, and 15 years post-transplant was 195, 148, 112, 88, 67, and 37, respectively.

**FIGURE 1:**
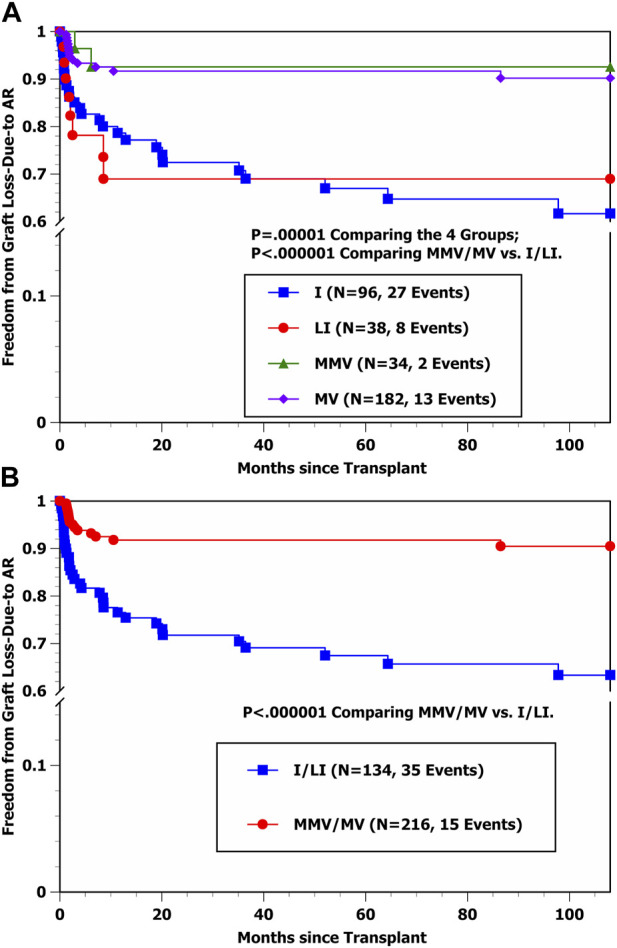
**(A)** Kaplan-Meier Freedom from graft loss-due-to AR by four transplant types (I, LI, MMV, and MV). **(B)** Kaplan-Meier freedom from graft loss-due-to AR by transplant type (I/LI combined vs. MMV/MV combined).

**FIGURE 2:**
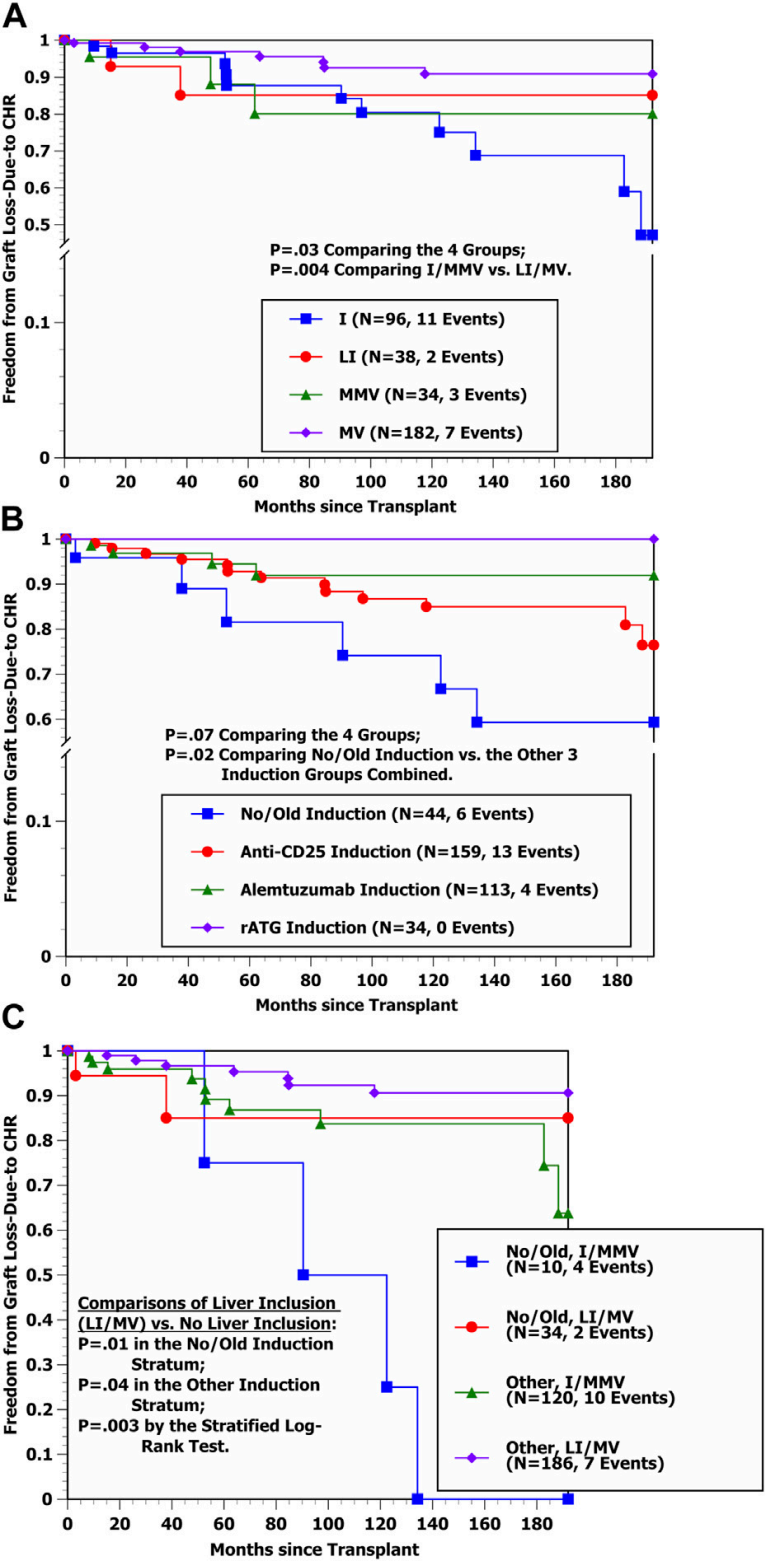
**(A)** Kaplan-Meier freedom from graft loss-due-to CHR by four transplant types (I, LI, MMV, and MV). **(B)** Kaplan-Meier Freedom from graft loss-due-to CHR by four induction groups (No/Old, anti-CD25, Alemtuzumab, and rATG). **(C)** Kaplan-Meier Freedom from graft loss-due-to CHR by induction group (No/Old vs. Other) and liver inclusion (No vs. Yes) (i.e., I/MMV vs. LI/MV).

Freedom from graft loss-due-to AR curves by transplant type in Figures 1A, B show that the hazard rate of graft loss-due-to AR was significantly higher among I and LI transplant cases in comparison with MMV and MV cases (*p* < 0.000001), with essentially identical outcomes for I vs. LI recipients as well as for MMV vs. MV recipients, respectively. Conversely, the freedom from graft loss-due-to CHR curves by transplant type in Figure 2A suggest that liver-containing (LI and MV) grafts had a more favorable outcome in comparison with liver-free (I and MMV) grafts (*p* = 0.002). Figure 2B shows that freedom from graft loss-due-to CHR was also less favorable for transplant recipients who received no/old induction in comparison with the other three induction groups combined (*p* = 0.02). Lastly, freedom from graft loss-due-to CHR curves by induction type (no/old vs. other) and transplant type (I/MMV vs. LI/MV) in Figure 2C clearly show a significantly more favorable outcome for liver-containing grafts once induction type was controlled (*p* = 0.01 in the no/old induction stratum; *p* = 0.04 in the other induction stratum; and *p* = 0.003 by the stratified log-rank test).

Using stepwise Cox regression, only one baseline variable was selected into the Cox model indicating a significantly lower hazard rate of developing graft loss-due-to AR (Table 4): Transplant Type MMV or MV (*p* < 0.000001). The estimated hazard ratio (HR) and 95% Confidence Interval (CI) for effect of Transplant Type MMV or MV was 0.240 [0.131–0.440]. Once this variable was controlled, none of other baseline variables offered additional prognostic value (*p* > 0.05). For instance, while Table 4 shows that Received Native Splenectomy, Received Donor Spleen, and Received Donor Liver (LI or MV) were each associated in univariable analysis with a significantly lower hazard rate of developing graft loss-due-to AR (*p* = 0.000002, 0.0006, and 0.003, respectively), due to their significant positive associations with Receiving Transplant Type MMV or MV, once this latter variable was controlled in the Cox model, multivariable tests to include Received Native Splenectomy, Received Donor Spleen, and Received Donor Liver (LI or MV) were non-significant (*p* = 0.91, 0.07, and 0.97, respectively).

**TABLE 4:** Cox model for the hazard rate of developing graft loss-due-to AR (50 events).

Selected Cox model via stepwise regression				
Baseline variable^a^	Univariable *p*-value	Multivariable *p*-value	Model Coeff ± SE	Estimated HR [95% CI]
Recipient Age	0.84			
Recipient Age ≥18 years	0.80			
Male Recipient	0.05			
Black (Non-Hispanic) Recipient	0.78			
Hispanic Recipient	0.55			
Intestinal Retransplant	0.70			
CMV Antibody Status: D+/R-	0.84			
Donor Age	0.09			
Transplant Type I	0.00003			
Transplant Type LI	0.06			
Transplant Type MMV	0.15			
Transplant Type MV	0.00007			
Transplant Type MMV or MV	<0.0000001	(√) <0.000001	−1.426 ± 0.309	0.240 [0.131–0.440]
Received Donor Liver (LI or MV)	0.003			
Received Donor Spleen	0.0006			
Received Donor Large Bowel	0.19			
Received Native Splenectomy	0.000002			
In Hospital Pretransplant	0.10			
Received No/Old Induction	0.03			
Received anti-CD25 Induction	0.18			
Received Alemtuzumab Induction	0.60			
Received rATG Induction	0.36			

Abbreviations: AR, acute rejection; Coeff, Coefficient; HR, hazard ratio; CI, confidence interval.

Note: (√) represents selection into the Cox model.

^a^ Variables included in the Cox model were defined as follows: Transplant Type MMV or MV = {1 if Transplant Type = MMV or MV, 0 otherwise}. Once Transplant Type MMV or MV was controlled, none of the other baseline variables offered additional prognostic (*p* > 0.05).

Using stepwise Cox regression, two baseline variables were selected into the Cox model indicating a significantly lower hazard rate of developing graft loss-due-to CHR (Table 5) (shown by order of selection): Received Donor Liver (LI or MV) (*p* = 0.002) and Received Induction Other than No/Old (*p* = 0.007). Estimated HRs and 95% CIs for the effects of Received Donor Liver (LI or MV) and Received No/Old Induction were 0.280 [0.119–0.661] and 3.379 [1.316–8.674], respectively. Once these two variables were controlled, none of the other baseline variables offered additional prognostic value (*p* > 0.05). Table 5 shows that while Transplant Type MMV or MV was associated in univariable analysis with a significantly lower hazard rate of developing graft loss-due-to CHR (*p* = 0.02), once the two selected variables were controlled, the multivariable test to include this variable yielded *p* = 0.94. Thus, the stepwise Cox model results in Tables 4, 5 match closely with the Kaplan-Meier comparisons shown in Figures 1A–2C.

**TABLE 5:** Cox model for the hazard rate of developing graft loss-due-to CHR (23 events).

Selected Cox model via stepwise regression				
Baseline variable^a^	Univariable *p*-value	Multivariable *p*-value	Model Coeff ± SE	Estimated HR [95% CI]
Recipient Age	0.19			
Recipient Age ≥18 years	0.42			
Male Recipient	0.42			
Black (Non-Hispanic) Recipient	0.49			
Hispanic Recipient	0.40			
Intestinal Retransplant	0.22			
CMV Antibody Status: D+/R-	0.90			
Donor Age	0.09			
Transplant Type I	0.009			
Transplant Type LI	0.99			
Transplant Type MMV	0.45			
Transplant Type MV	0.006			
Transplant Type MMV or MV	0.02			
Received Donor Liver (LI or MV)	0.004	(√) 0.002	−1.272 ± 0.438	0.280 [0.119–0.661]
Received Donor Spleen	0.45			
Received Donor Large Bowel	0.95			
Received Native Splenectomy	0.05			
In Hospital Pretransplant	0.97			
Received No/Old Induction	0.02	(√) 0.007	1.218 ± 0.481	3.379 [1.316–8.674]
Received anti-CD25 Induction	0.85			
Received Alemtuzumab Induction	0.21			
Received rATG Induction	0.27			

Abbreviations: CHR, chronic rejection; Coeff, Coefficient; HR, hazard ratio; CI, confidence interval.

Note: (√) represents selection into the Cox model.

^a^ Variables included in the Cox model were defined as follows: Received Donor Liver (LI or MV) = {1 if Transplant Type = LI or MV, 0 otherwise}; and Received No/Old Induction = {1 if Recipient received No/Old Induction, 0 otherwise}. The order of selection for the two baseline variables selected into the Cox model via stepwise regression were as follows: Received Donor Liver (LI or MV), and Received No/Old Induction. Once the two selected variables were controlled, none of the other baseline variables offered additional prognostic (*p* > 0.05).

## Discussion

The results of this observational study with a median follow-up of nearly 13 ½ years post-transplant demonstrate three findings: 1) Transplant Type MMV or MV is the single factor that clearly protects against graft loss-due-to AR, whereas this combination of transplant types did not independently protect against graft loss-due-to CHR, 2) Once the favorable influence of Transplant Type MMV or MV on the hazard rate of graft loss-due-to AR was controlled, Liver Inclusion (Transplant Type LI or MV) showed no protective effect against graft loss-due-to AR, and 3) Liver Inclusion appears to independently and significantly protect against graft loss-due-to CHR. These results are consistent with our most recent report [8] (as well as with our earlier reports [9–12]) showing that Transplant Type MMV or MV but not Liver Inclusion protects against the development of a first ACR (of any grade) [8] as well as against the development of a severe ACR [8–12]. At our center, Transplant Type MMV or MV is nearly completely distinguished from Transplant Type I or LI by the joint removal of the native pancreaticoduodenal complex and native spleen; thus, the extensive removal of native lymphoid tissue (i.e., spleen, mesenteric lymph nodes, and intestinal mucosal lymphoid tissue) would appear to explain the more favorable freedom from ACR, severe ACR, and graft loss-due-to AR outcomes that we have observed over the years for Transplant Type MMV or MV.

Such a scenario was also shown in a cardiac allograft animal model with indefinite immunological tolerance after removal of secondary lymphoid organs [24]. Conversely, a separate cardiac allograft animal study from the University of Pittsburgh showed that while liver inclusion did not protect against subsequent ACR incidence, it provided clear protection against the development of CHR [25]. Previous intestinal transplant results by the University of Pittsburgh have also demonstrated a clear protective effect of Liver Inclusion against the development of CHR [6, 15]; thus, the CHR results reported here are, in fact, consistent with the University of Pittsburgh findings. It should also be noted that in none of their earlier studies [2, 5, 6] (to our knowledge) were any multivariable analyses of the hazard rates of developing a first ACR, severe ACR, or graft loss-due-to AR ever reported.

The vast vascular (sinusoidal) endothelial surface of the liver uniquely enables it to absorb circulating DSAs, thereby offering protection against potential acute and chronic damage caused by their presence [26]. This type of protection is similarly offered in both liver-alone and liver-combined-with other organ transplants (e.g., liver-kidney, liver-heart) [26]. In kidney-alone transplants, it is well-known that the presence of DSAs are associated with significantly higher rates of developing hyperacute rejection, ACR, and acute AMR [27–31], and studies of simultaneous liver-kidney transplantation have clearly demonstrated protection by liver inclusion against these types of rejection [32]. In intestinal transplantation, liver inclusion has been shown to be helpful in clearing preformed DSAs [16] as well as to offer protection against the development of *de novo* DSAs [16, 33–35]. Abu-Elmagd et al [16] also showed that while persistent performed and *de novo* DSAs were significantly associated with a much higher hazard rate of developing CHR, no significant associations of these types of DSAs with the hazard rate of developing ACR were observed. In fact, the hazard rates of developing ACR and CHR were not noticeably different between recipients having preformed DSAs that cleared after transplant vs. those who remained free of DSAs both before and after transplant [16]. Kubal et al [33] also appeared to show associations between the presence of *de novo* DSAs and higher rates of developing acute AMR and CHR but without a concomitantly higher rate of developing ACR (note: a clear distinction was made in that study between acute AMR presence vs. strictly ACR occurrence). Other previous studies have reported an association between the presence of *de novo* DSAs and a higher incidence of ACR development, but without a clear separation of acute AMR presence vs. strictly ACR occurrence being made [36, 37]. Thus, while it is still unclear as to what extent liver inclusion offers protection against the potential damage of circulating DSAs in intestinal transplantation, the results presented to date do indicate a clear protection of its inclusion against CHR development.

Since DSA and humoral rejection data were not available in most of our patients transplanted prior to 2013, no attempt to analyze such results was made here, which is a clear study limitation. In addition, while Wu et al [17] showed that the presence of DSAs were associated with a significantly higher risk of developing acute AMR, with liver-containing allografts offering significant protection against acute AMR development, no standardized definition of acute AMR has yet to be made in intestinal transplantation.

Another clear study limitation was the fact that this study spans over several years, and variables such as immunosuppression, indications, and surgical techniques have changed. This makes interpretation of the results rather difficult. However, multivariable analysis of predictors of the hazard rate of graft loss-due-to AR found no significant effects of induction type, and multivariable analysis of predictors of the hazard rate of graft loss-due-to CHR found that only our earliest approaches (“no/old induction” during 1994–1997) were significantly less favorable. In addition, while this cohort of 350 consecutive intestinal transplant cases were prospectively followed and represents one of the largest experiences with intestinal transplantation ever reported, the liver-intestine and modified multivisceral subgroups were relatively small. Thus, generalization of our results to other centers could be limited by these relatively small subgroup sample sizes. Nonetheless, we believe that we are reporting statistically sound results regarding the significant multivariable predictors of the hazard rates of developing graft loss-due-to AR vs. CHR.

Other observed differences in clinical outcomes between two of the historically largest intestinal transplant centers are worth noting. As reported here, among the transplanted cases who experienced graft loss-due-to rejection, 68.5% (50/73) and 31.5% (23/73) were due to AR and CHR, respectively. This is in stark contrast to the University of Pittsburgh results (with similarly long patient follow-up) [6] in that only 25.7% (26/101) of their reported graft losses due-to-rejection were due to AR, whereas 74.3% (75/101) were due to CHR. In terms of absolute numbers, the observed percentages who developed graft loss-due-to AR and CHR in this study were 14.3% (50/350) and 6.6% (23/350), respectively, versus 5.2% (26/500) and 15.0% (75/500) in the Abu-Elmagd et al study [6], similar in value when the two outcomes are combined. However, severe ACR usually occurs much earlier post-transplant in comparison with CHR occurrence. Since nearly all patients at our center who experienced graft loss-due-to AR had previously experienced a severe ACR [8–12], is it possible that the (unreported) incidence rate of severe ACR was concomitantly lower among University of Pittsburgh patients who received a preconditioning anti-lymphocyte induction regimen [6] with either rATG (thymoglobulin) or alemtuzumab (in comparison with our historical cohort of 350 patients)? Is it possible that their preconditioning strategy to give most (or all) of their anti-lymphocyte induction prior to reperfusion [38–40] helps to alleviate severe ACR risk? These questions are still left unanswered.

We also recently reported more favorable graft survival outcomes using our newer, more intensive induction strategy (since 2013) of combining a larger total dose (post-reperfusion) of rATG (10 mg/kg, 2 mg/kg ×5) with 1 standard rituximab dose given during the first 8 days post-transplant (with longer prophylactic care as well) [8, 41]. In our most recent report [8], fewer ACR (of any grade) and severe ACR episodes were observed among the 95 patients who received this more intensive rATG/rituximab induction strategy, with the observed percentages developing graft loss-due-to AR and CHR during the first 60 months post-transplant being 7.4% (7/95) and 3.2% (3/95), respectively. It will therefore be of interest to recalculate these percentages with more patients and after longer post-transplant follow-up has accrued.

In summary, while the results reported here are based on an historical cohort of intestinal transplant cases who were transplanted at our center between 1994–2012 and received varying older induction immunosuppression protocols, we believe this study has helped to clarify some of the previously reported discrepancies in results that have existed between our center and the University of Pittsburgh regarding predictors of graft loss-due-to AR vs. graft loss due to CHR. It is our hope that some additional clarity has been provided here in terms of distinguishing between these two important clinical outcomes following intestinal transplantation. In addition, while direct comparison of two high volume intestinal transplant programs is relevant, it begs the question of a collaborative investigation using a multi-center approach rather than independent reports.

## Data Availability

The raw data supporting the conclusion of this article will be made available by the authors, without undue reservation.

## Abbreviations

ACR, acute cellular rejection; AMR, antibody-mediated rejection; AR, acute rejection; CHR, chronic rejection; DSA, donor specific antibody; I, isolated intestine; LI, liver-intestine; MMV, modified multivisceral; MV, multivisceral; mo, months; rATG, rabbit antithymocyte globulin; SE, standard error; TAC, tacrolimus; wk, week.
